# Psychometric validation, factor structure, and measurement invariance of the Arabic brief existential loneliness scale

**DOI:** 10.3389/fpsyg.2026.1804096

**Published:** 2026-06-09

**Authors:** Khaled Awad Al-Dassean

**Affiliations:** Al-Balqa Applied University, Al-Shoubak University College, Al Shoubak, Jordan

**Keywords:** Arabic version, existential, isolation, loneliness, measurement invariance, psychometric validation

## Abstract

**Introduction:**

Existential loneliness signifies a profound feeling of fundamental separation from others and the world, increasingly acknowledged in the scholarly literature. The Arabic psychological literature lacks the necessary instruments to assess this construct.

**Methods:**

The objective of the current study was to develop an Arabic version of the Brief Scale of Existential Loneliness (BSEL). Psychometric validation included internal consistency, test–retest reliability, and validity. The factor structure of the scale was assessed using exploratory factor analysis and confirmatory factor analysis with 420 participants (aged 18–82 years; M = 35.7, SD = 15.3), followed by assessing measurement invariance across gender and age groups.

**Results:**

The results showed excellent internal consistency reliability (alpha and omega) and adequate stability. BSEL scores had a positive correlation with depression and existential isolation and a negative correlation with happiness, thus indicating convergent–divergent validity of the scale. The BSEL score showed a negative correlation to age. A unidimensional factor structure was supported. Strict measurement invariance was established across genders, whereas scalar invariance was supported across age groups.

**Discussion:**

The results suggest that the Arabic version of the BSEL is a promising and user-friendly tool for assessing existential loneliness among native Arabic speakers.

## 1 Introduction

A considerable percentage of the global population experiences distressingly elevated levels of loneliness, with the incidence among adolescents in Eastern Mediterranean nations at 14.4% and among older adults in Eastern European countries varying from 18.7 to 24.2% (Surkalim et al., 2022). Loneliness constitutes a significant crisis in the contemporary world, underpinning the challenges people face (Qashqoush, 1983). It exerts an equivalent influence on health and longevity as other clinical risk factors (Pantell et al., 2013), is associated with depression (Cacioppo et al., 2006; Fried et al., 2015), and results in the decline of daily activities and motor performance, and a heightened need for specialized nursing facilities (Perissinotto et al., 2012; Pomeroy et al., 2023). Loneliness is also associated with reduced trust in others, since lonely people exhibit lower levels of empathy, resulting in decreased social cohesion (Cuccu and Stepanova, 2021). As a result, there has been an increasing clamor to make loneliness a public health priority. For example, the European Union has developed a strategy that addresses the mental health issues associated with loneliness (Schnepf et al., 2024, p. 6). Existential loneliness is a phenomenon that is finding greater recognition in the literature (Mayers and Svartberg, 2001; Mayers et al., 2002; Ettema et al., 2010; Yalom, 1980). Nonetheless, it was undermined by ambiguity, with the term being employed either interchangeably or independently in the literature (Álvarez et al., 2023). To understand existential loneliness and be able to distinguish between its different aspects, as asserted by Ettema et al. (2010), it is essential to differentiate between three commonly used terms in existential literature: isolation, solitude, and loneliness. “Isolation” refers to the actual state of separation, which is an external objective condition that can often be controlled. When an individual perceives it, it transforms into an internal experience, with two possible paths: it can take the form of solitude, which represents the positive aspect of isolation, or it can take the form of loneliness, a painful and distressing experience.

Existential loneliness, as viewed by many theorists of the existential school, is an intrinsic human condition (Moustakas, 1961). In their opinion, this feeling is considered an inevitable state from which one cannot escape (Mayers and Svartberg, 2001). It involves a cognitive-perceptual element, resulting from the individual’s feeling and perception of the gap between their actual relationships with others and their desire for such interactions (Hopps et al., 2001; De Jong Gierveld, 1998). According to Bolmsjö et al. (2018), five fundamental factors contribute to existential loneliness: imminent death and its thoughts, physical disability, fear of disease recurrence, inability to communicate, and the feeling that others do not acknowledge our need to express ourselves, nor understand us. Consequently, it goes beyond the issue of deficiency in interpersonal, social, and emotional relationships (Saricali and Guler, 2022), reaching a profound depth of feelings of fundamental separation from others and the world, leading individuals to perceive that their experiences cannot be fully shared by anyone (Pinel et al., 2017). It is accompanied by sensations of isolation, emptiness, and lack of purpose (Larsson et al., 2019), and is challenging to address through companionship and or an expanded network of relationships (Mayers et al., 2002). However, it is commonly associated with aging (McKenna-Plumley et al., 2024), although anyone can experience a degree of existential loneliness (Mayers and Svartberg, 2001). Qualitative research has been conducted in the domains of palliative care and nursing facilities (Chung et al., 2020), severe illnesses (Tarbi and Meghani, 2019; Philipp et al., 2021; Baglow et al., 2024), gerontology (Carr and Fang, 2023; Larsson et al., 2019), end-of-life scenarios and mortality (Boston et al., 2011; Rosedale, 2007; Ettema et al., 2010), among immigrants (Olofsson et al., 2021), and frail elderly individuals (Sjöberg et al., 2017). In a recent qualitative study, Garnow et al. (2024) examined the experience of existential loneliness during adolescence, describing its distinctive characteristic as a feeling of being lost, trapped between belonging and self-discovery. However, there is a dearth of information on the prevalence and nature of existential loneliness among different demographic groups in Arab countries, including Jordan. Nor are there any quantitative surveys on the subject. The primary explanation appears to be the lack of a valid measurement tool on this subject. The increasing evidence from studies linking existential loneliness to psychological disorders such as depression, anxiety, and suicidal thoughts (Kretschmer and Storm, 2018; Saricali and Guler, 2022; Kleftaras and Psarra, 2012) underscores the urgency for creating new measurement instruments grounded in robust conceptual frameworks. These tools should effectively capture this phenomenon, enable researchers to investigate its effects on various aspects of human life, and facilitate its diagnosis by clinical practitioners who adopt existential therapy (Gökdemir-Bulut and Bozo, 2018).

Many researchers attribute the dearth of studies on existential loneliness to the lack of valid and reliable measurement tools (Hadeei, 2023; McKenna-Plumley et al., 2024) capable of differentiating existential loneliness from other forms, such as social and emotional loneliness, thereby recognizing it as a distinct conceptual construct. This underscores the necessity of developing instruments to assess existential loneliness and evaluating their psychometric characteristics, as the absence of such tools is a significant challenge for researchers in Eastern cultures, particularly among Arabic-speaking populations. In fact, only a few existential loneliness scales have been developed as a specific trait recognized in the literature (Van Tilburg, 2020), and to our knowledge, their number does not exceed five. One of them is antiquated—produced over 50 years ago—lengthy, conceptually intricate, impracticable, infrequently utilized in research, and has encountered substantial criticism (Gökdemir-Bulut and Bozo, 2018; Hadeei, 2023; Mckenna-Plumley et al., 2024). It comprises a subscale of eight items designed to assess existential loneliness among a total of 60 items aimed at measuring general loneliness; it is the Extended Loneliness Scale (BELS; Belcher, 1973). The second scale is the Existential Loneliness Questionnaire (ELQ; Mayers et al., 2002), developed in a small sample of women with acquired immune deficiency syndrome. The third tool is the Existential Isolation Scale (EIS; Pinel et al., 2017), a concise 6-item scale that assesses the cognitive dimension of loneliness, excluding emotional-affective components. The fourth scale, the Existential Loneliness Scale (ELS), comprises 19 items, built on the characteristics of loneliness, its causes, interrelated factors, and the circumstances under which it occurs. It is internally consistent and has stability over a month (Hadeei, 2023). The fifth scale is the Brief Scale of Existential Loneliness (BSEL; Mckenna-Plumley et al., 2024). This is a new scale that has not been tested in other cultures or had its psychometric properties checked since the first study. It seems to be one of the most promising scales in the field, as it was built on solid conceptual foundations. The Arabic version of the BSEL scale provides an important basis for identifying groups most susceptible to psychological distress and enhances early preventive efforts (Van Orden et al., 2010). The literature on loneliness indicates that integrating a measure of existential loneliness into clinical assessment helps uncover the deeper dimensions of psychological distress associated with self-isolation and loss of meaning, which traditional measures of loneliness may not adequately capture (Pinel et al., 2017). At the therapeutic level, diagnosing existential loneliness contributes to guiding and improving meaning-based interventions within the framework of existential therapies, which are noted for their effectiveness in reducing feelings of loneliness and enhancing psychological well-being (Wong, 2010; Vos et al., 2015). Therefore, offering a practical and concise tool for measuring existential loneliness represents an added value in integrated clinical practice. The set of existential loneliness scales currently available (ELQ, ELS, BSEL, EIS) reveals a promising field characterized by the development of various psychological measurement tools, though predominantly constructed within Western cultural frameworks, with just one recent addition originating from an Eastern, Persian context (ELS). These scales have demonstrated excellent internal consistency and good construct validity (e.g., the BSEL scale), indicating that existential loneliness, despite its precision and conceptual complexity (Álvarez et al., 2023), may be measured correctly. The aforementioned cross-cultural adaptations of these tools in Turkish, Iranian, Chinese, German, and Russian contexts have successfully shown their reliability. Most exploratory and confirmatory factor analysis studies supported the unidimensionality of the existential loneliness trait (e.g., Hadeei and Aminikhou, 2023; Hadeei, 2023; Mckenna-Plumley et al., 2024), thus augmenting the construct validity of these scales. However, a study by Gökdemir-Bulut and Bozo (2018) showed a multidimensional structure for the Turkish version of the ELQ scale, which Hadeei (2023) attributed to an issue with appropriateness of the statistical analysis method. The construct validity was supported through concurrent and convergent validity, which exhibited consistent correlations with closely related psychological variables, such as depression and anxiety (Hadeei, 2023). It indicated discriminant validity through consistent negative associations with various psychological measures, including well-being and meaning in life (Hadeei and Aminikhou, 2023). Moderate strength correlations with general loneliness scales somewhat supported its discriminant validity in distinguishing general loneliness from existential loneliness (Mckenna-Plumley et al., 2024), enhancing the utility of these scales in survey and clinical research pertaining to existential loneliness. Measurement invariance has not been comprehensively established across cultures, except for one cross-cultural study (Zhou et al., 2023) on the Existential Isolation Scale (EIS) between German and Chinese samples, which showed measurement invariance, highlighting the need for more studies to confirm measurement invariance across diverse cultures.

Despite the continuous improvements in these measures based on qualitative and quantitative studies, the issue of conceptual fragmentation, as described by Álvarez et al. (2023) in the operationalization of the concept of existential loneliness, continues to hinder and complicate the comparison and integration of these measures. However, in Jordan, multiple studies have been undertaken to assess concepts related to existential loneliness, such as the study by Alfuqaha et al. (2022) for the translation and adaptation of the existential scale. Additionally, a study conducted by Musa (2014) aimed to validate the existential well-being scale. A separate study concerning spiritual well-being and quality of life in dialysis patients in Jordan aimed to differentiate between the theological and existential aspects of spiritual well-being (Musa et al., 2023). One qualitative study examined the experience of existential loneliness among Arab immigrant women in Sweden (Olofsson et al., 2024). The aforementioned research suggests that the conceptual framework of existential loneliness is examined and comprehended within the Arab cultural context and is functional; yet, unlike in Western cultures, it lacks the requisite tools for a thorough exploration of this problem. To the best of the researcher’s knowledge, the Arab world, particularly Jordan, has not developed or translated any specific instruments for measuring existential loneliness.

### 1.1 The current study

The psychological literature related to existential loneliness indicates that it is a largely unexplored field, especially in collectivist societies (Chung et al., 2020). To the best of our knowledge, there is an absence of measurement tools for existential loneliness in Arab culture as a whole. Considering the importance of issues related to existential loneliness as a global concern, we found it useful to study the psychometric properties of existential loneliness scales and their cross-cultural adaptation for Arabic speakers. Our goal was to bridge this gap by introducing a contemporary global scale for existential loneliness, the Brief Scale of Existential Loneliness (BSEL; McKenna-Plumley et al., 2024; 6 items), which offers the advantages of a brief survey. To evaluate its suitability for Arab culture, we first translated it into Arabic and then assessed its psychometric properties, structural validity, and measurement invariance across gender and age. Based on the original study, we expected the scale to provide a good fit for the unidimensional model with adequate internal consistency. We expected there to be positive relationships between BSEL and both depression and existential isolation, while negative connections were expected with happiness. We held no expectations regarding the findings of the measurement invariance analysis, owing to the scarcity of research on this issue within Arab culture. The objective was to investigate whether the psychometric meaning of the assessed constructs is consistent across gender and age demographics.

## 2 Methods

### 2.1 Study design

A cross-sectional study was carried out in three phases, including a pilot sample and two subsamples. Phase one: Translation and Pilot Study proceeded with the pilot sample (*n* = 35). Phase two (Sub-sample 1, *n* = 220): The aim was to evaluate convergent and divergent validity, in addition to doing exploratory factor analysis. Phase Three (Sub-sample 2, *n* = 200): The aim was to conduct confirmatory factor analysis to assess construct validity. A multi-group confirmatory factor analysis was conducted on the entire study sample (*N* = 420) to assess measurement invariance.

### 2.2 Participants

The 420 study participants (64.5% female, *n* = 271), aged 18 to 82 years (M = 35.7, SD = 15.3), were divided into two sub-samples. Sub-Sample 1 for exploratory factor analysis and validity consisted of 220 participants (74.5% female, *n* = 164), aged 18 to 75 years (M = 38.5, SD = 14.5). Sub-sample 2 constituted a confirmatory factor analysis group involving 200 participants (53.5% female, *N* = 107), aged from 18 to 82 years (M = 32.8, SD = 7.15). Table 1 presents a comprehensive picture of the characteristics of the sample.

**Table 1:** Demographic characteristics of the study samples.

Variable	level	Sub-sample 1 (*N* = 220)	Sub-sample 2 (*N* = 200)	Whole sample (*N* = 420)
Gender N (%)	Male	56 (25.5%)	93 (46.5%)	149 (35.5%)
	Female	164(74.5%)	107 (53.5%)	271 (64.5%)
Age M (STD) Range	Year	(14.5) 38.5Range 57	32.7 (15.6) Range 64	35.7 (15.3)64
Academic level N (%)	Secondary	22 (10%)	16 (8%)	65 (15.5%)
	Diploma	40 (18.2%)	48 (24%)	78 (18.6%)
	Bachelor	145 (65.9%)	136 (68%)	264 (62.8%)
	Master/PhD	13 (5.9%)	-	13 (3.1%)
Marital status N (%)	Single	90 (40.9%)	128 (64%)	217 (51.7%)
	Marriage	124 (56.3%)	72 (36%)	197 (46.9%)
	Divorced	1 (0.5%)	-	1 (0.2%)
	Widow	5 (2.3%)	-	5 (1.2%)
Sample kind N (%)	Student	92 (41.8%)	119 (59.5%)	214 (51%)
	General public	(%58.2) 128	81(40.5%)	206 (49%)

### 2.3 Measures

#### 2.3.1 Brief scale of existential loneliness

McKenna-Plumley et al. (2024) developed the BSEL in the UK and Ireland to address the lack of instruments for assessing existential loneliness, especially brief versions, after a comprehensive review and qualitative study. The scale is applicable to several age groups, from youth to the elderly. Unidimensional and with six items, it focuses on the self-reported suffering associated with existential loneliness. The response level ranges from 1 to 5 on a Likert scale, where 1 indicates strong disagreement and 5 strong agreement (e.g., “I struggle with the feeling that I am separate from other people”). The scale has strong internal consistency (alpha = 0.94, McDonald’s omega = 0.94) and adequate construct validity, as determined by known-groups validity, efficiently distinguishing between individuals with severe mental health issues and those in borderline conditions. The scale shows a negative association with the meaning in life construct and a positive correlation with general loneliness, poor mental health, and other related constructs.

#### 2.3.2 Existential isolation scale

An Arabic version of the EIS scale (Pinel et al., 2017; currently under study) was utilized, having undergone identical translation and validation processes, along with the pilot study of the BSEL, due to the absence of previous equivalent instruments in Arabic for assessing existential isolation. It is part of a continuous research project. The scale focuses on the cognitive aspect of existential isolation, emphasizing that an individual’s life experience is fundamentally different from that of others. The scale consists of six items: four positive items (items 1, 2, 3, and 6) whose scores are reversed when calculating the individual’s total score, and two negative items (items 4 and 5; e.g., “I usually feel like people share my outlook on life”). They are answered using a 10-point Likert scale ranging from 0 (strongly disagree) to 9 (strongly agree). A high total score on the scale reflects a strong sense of existential isolation. The unidimensional scale is characterized by good internal consistency (alpha = 0.83). The properties of the scale were studied using diverse samples such as Russian (Smirnov et al., 2025), German and Chinese (Zhou et al., 2023), and South Korean (Park and Pinel, 2020), where it showed good reliability and validity indicators. We chose this scale because of its substantial association with existential loneliness, and we expected a moderate to high correlation with BSEL to reveal convergent validity. The Cronbach’s alpha value indicated high reliability (*α* = 0.91) in the current study.

#### 2.3.3 Depression anxiety stress scale (DASS-21)

The Arabic version of the depression subscale of the DASS-21 (Al-Dassean and Murad, 2024) was used. This self-administered questionnaire consists of 21 items distributed across three related but distinct subscales (depression, anxiety, and stress), with seven items for each. The depression subscale evaluates depressive symptoms such as decreased life satisfaction, self-esteem, loss of hope, and lethargy (e.g., “I felt that life was meaningless”). Participants’ responses are measured on a 4-point Likert scale ranging from 0 (not applicable) to 3 (frequently applicable). Higher scores indicate higher levels of depressive symptoms, with the score on the depression subscale ranging from 0 to 28. The scale has sufficient indicators of validity and reliability; in the current study, the Cronbach’s alpha reliability coefficient was 0.85.

#### 2.3.4 The Arabic happiness scale

The scale consists of 15 concise items, including five psychologically relevant filler items that are excluded from the total score (Abdel-Khalek, 2013). Responses to the scale are given using five levels of response: 1 (not at all) to 5 (very high; e.g., “my life has meaning”). The total score ranges from 15 to 75, and as the score on the scale increases, it indicates a higher level of happiness for the individual. It includes two sub-scales: general happiness and successful life. The Cronbach’s alpha reliability values on different samples range between 0.90–0.94, and the test–retest reliability between 0.82–0.90. Construct validity was assessed in relation to recognized global measures, including the Fordyce Happiness Scale, the Self-Happiness Scale, and the Oxford Happiness Inventory, exhibiting correlations with external criteria such as overall health and life satisfaction. The scale possesses good psychometric properties and has an equivalent English version. It is worth mentioning that the five filler items (items 3, 6, 8, 14, and 17) were included to control for systematic response bias and do not contribute to the score on the scale. The Cronbach’s alpha value in the current study sample was 0.82.

### 2.4 Procedure

Participants were recruited through online advertisements on social media, student groups attached to Al-Balqa Applied University (specifically via WhatsApp), and the official university email for students and faculty members. For the general public, recruitment was done through social media platforms such as Facebook, Instagram, and WhatsApp. The only inclusion criterion was that the participant must be 18 years or older.

The convenience sampling method and snowball sampling were used to reach the largest possible number of participants. The data was collected by electronic questionnaires created with Google Forms, and the link to these questionnaires was shared via email, WhatsApp, Facebook, and other platforms. Participants were also asked to share the link with their contacts and peers. The survey for the retest was completed in paper format within the classroom, in the presence of the researcher. Students enrolled in the Measurement and Evaluation and Learning Theories courses at Al-Shobak University College (*N* = 42) were asked to identify themselves using a code or a distinctive mark of their choice without revealing their identities in the first application and to rewrite it in the second application to ensure response matching and maintain confidentiality. Seven students did not attend the second session, resulting in a total of 35 students participants’.

The study was conducted in accordance with the Helsinki Declaration and received approval from the Scientific Research Committee of Al-Shoubak University College. All participants were volunteers who received no money or incentives. They were told they could join or leave the study whenever they wanted. Participants were told of the study’s objective, guaranteed the confidentiality of their information, and assured that the data would only be utilized for scientific research purposes. Informed consent was obtained electronically prior to the start of the survey. The data collection process continued from September to November 2025.

#### 2.4.1 Additional procedures for adapting BSEL

Following contact the original author of the BSEL, permission was granted to develop an Arabic parallel version. The International Test Commission (ITC, 2017) reference manual for test preparation and cross-cultural adaptation was followed too. The scale was translated into Arabic by a specialized English–Arabic translator. Concurrently, the researcher independently translated another preliminary version of BSEL. The two versions were compared, resulting in a combined preliminary version of the translation. The unified translation was provided to a clinical psychology expert, a native Arabic speaker fluent in English, to perform the back translation without prior knowledge of or access to the original text. The translators and the researcher collaborated to analyze the back-translation alongside the original text. The degree of alignment between the two versions in psychological meaning, rather than the literal wording, was discussed. This comparison indicated that the psychological meanings and connotations between the two texts were aligned and that the existential concepts were accurately conveyed. After that, the initial translation of the tool was presented for review along with the original text to five faculty members at Jordanian universities, including two specialists in psychological measurement, two clinical psychologists, and one Arabic language expert. Minor modifications, primarily linguistic, were implemented to the phrasing of two items to conform to Modern Standard Arabic. The final step involved pilot testing of the final unified version of BSEL on a sample of 25 participants, comprising 15 university students and 10 members of the general public, to ascertain the clarity of the questions and the response instructions. The researcher also ensured that the text was free from any expressions that might conflict with Arab culture and provoke non-cooperation from the respondents. Thus, this application provided some support for the content validity of the scale and its readiness for undergoing psychometric validation.

### 2.5 Statistical analyses

All statistical analyses were conducted using SPSS software version 23 and JASP software version 0.95.4.0. Before beginning the analysis, the data was examined for any missing values. The percentage of missing cases was minimal, less than 4% (3 out of 420), and this missing data was notable in sub-sample 1. No indicators of a systematic missing data mechanism were observed; in light of this, the listwise deletion method was used, which is considered appropriate because it does not reduce statistical power or affect validity of the results (Little and Rubin, 2019). We used the conventional method for estimating the valid sample size for factor analysis based on the rule of 10–20 participants per item (totaling 60–120 participants). However, we exceeded this number; our sample sizes were 220 for Exploratory Factor Analysis (EFA) and 200 for Confirmatory Factor Analysis (CFA). Descriptive statistics for the translated BSEL scale were also computed.

In line with the optimal methodology for scale adaptation, especially if the theoretical construct being investigated is culturally related, as was the case with existential loneliness in the current study, we chose to perform an exploratory factor analysis (Borsa et al., 2012). The principal axis method and parallel analysis were employed to determine the number of retained factors (Fabrigar et al., 1999), alongside the Kaiser-Guttman criterion, eigenvalues exceeding 1, and the explained variance ratio (Hair et al., 2019). An item is retained if its loadings on the factor exceed 0.4 and do not cross-load on other factors by more than 0.32 (Worthington and Whittaker, 2006). Reliability was estimated through internal consistency using Cronbach’s alpha (*α*), McDonald’s omega (*ω*), and the test–retest method with a 3-week interval between the first and second administrations, computing the correlation (r) between the two administrations.

A CFA was performed to verify the factor structure revealed by the EFA. Model modifications were guided by both statistical and theoretical considerations. Specifically, the corrected error terms were added only when they were statistically conditioned by modification indices and theoretically justified based on the content of the items. Before estimating the model, the data were assessed for compliance with the assumption of multivariate normality using Mardia’s test (Mardia, 1970), which demonstrated significant results (skewness χ^2^(56) = 260.44, *p* < 0.001, and kurtosis z = 14.86, *p* < 0.001), indicating a significant violation of this assumption. Consequently, the model parameters were estimated using maximum likelihood mean and variance adjusted (MLMV) with robust standard errors, which provides the best combination of standard errors and Type I errors with non-normal data (Maydeu-Olivares, 2017). Several goodness-of-fit indices were employed to evaluate the model, including the relative/normed chi-square (χ^2^/df) due to the traditional chi-square’s sensitivity to sample size and its assumption of multivariate normal distribution. Consequently, increased emphasis was placed on incremental fit indices, including the Comparative Fit Index (CFI), Tucker–Lewis Index (TLI), Standardized Root Mean Square Residual (SRMR), and Root Mean Square Error of Approximation (RMSEA). The model had a good fit with X^2^/df < 3, CFI and TLI ≥ 0.95, and SRMR and RMSEA ≤0.08 (Dimitrov, 2012; Hooper et al., 2008). Bivariate correlation coefficients were computed between BSEL scores and the Existential Isolation Scale (EIS), as well as the depression subscale of the DASS-21, to assess convergent validity. The positive associations were to be expected, given that the feeling of existential isolation leads to existential loneliness (McKenna-Plumley et al., 2024). Divergent validity was assessed by the correlation coefficients between BSEL scores and the Happiness Scale (ASH). Discriminant validity was assessed with the HTMT approach. Since an HTMT value close to 1 indicates a lack of discriminant validity, the HTMT value was compared to a more conservative predetermined cutoff point of 0.85. If the value exceeds 0.85, it indicates a lack of discriminant validity (Henseler et al., 2014). Measurement invariance was established across gender and age groups by Multi-Group Confirmatory Factor Analysis (MG-CFA) with a hierarchical sequence of model constraints: configural, metric, scalar, and residual invariance. The changes in fit indices between successive models were utilized, with values not exceeding ΔCFI ≥ − 0.005, ΔRMSEA ≤ 0.01, and ΔSRMR ≤ 0.005, and the metric ΔSRMR ≤ 0.025 was considered an indicator of the model’s goodness of fit (Chen, 2007).

## 3 Results

### 3.1 Descriptive statistics

Descriptive statistics for A-BSEL items were computed, including the mean, standard deviation, skewness, and kurtosis, in line with the criterion established by Tabachnick and Fidell (2007), which suggests that if the skewness and kurtosis values for the items range from −1.5 to 1.5, the data follows a univariate normal distribution. Based on this criterion, the data from the two sub-samples and the whole sample all followed a univariate normal distribution. Upon testing the data for the assumption of multivariate normality through Mardia’s test, all results were significant, suggesting that the data did not conform to a multivariate normal distribution. Table 2 shows these findings.

**Table 2:** Descriptive statistics and CFA factor loadings (β) for scale items (Sub-sample 2).

Item	Sub-sample 1		Sub-sample 2		Whole sample			CFA results
	S	K	S	K	S	K	M (STD)	Factor loading (β)
1	0.18	−0.78	0.56	−0.49	0.36	−0.69	2.40 (1.1)	0.759
2	0.43	−0.71	0.69	−0.58	0.54	−0.70	2.39 (1.0)	0.766
3	0.33	−0.69	0.82	0.19	0.56	−0.35	2.29 (1.1)	0.851
4	0.44	−0.66	0.74	−0.48	0.58	−0.60	2.24(1.1)	0.808
5	0.51	−0.60	0.86	−0.24	0.69	−0.42	2.14(1.1)	0.795
6	0.69	−0.37	1.10	0.26	0.88	−0.12	2.02(1.1)	0.861

S, Skewness; K, kurtosis; M, Mean; STD, Standard deviation; CFA, Confirmatory factor analysis.

### 3.2 Exploratory factor analysis

**Figure 1:**
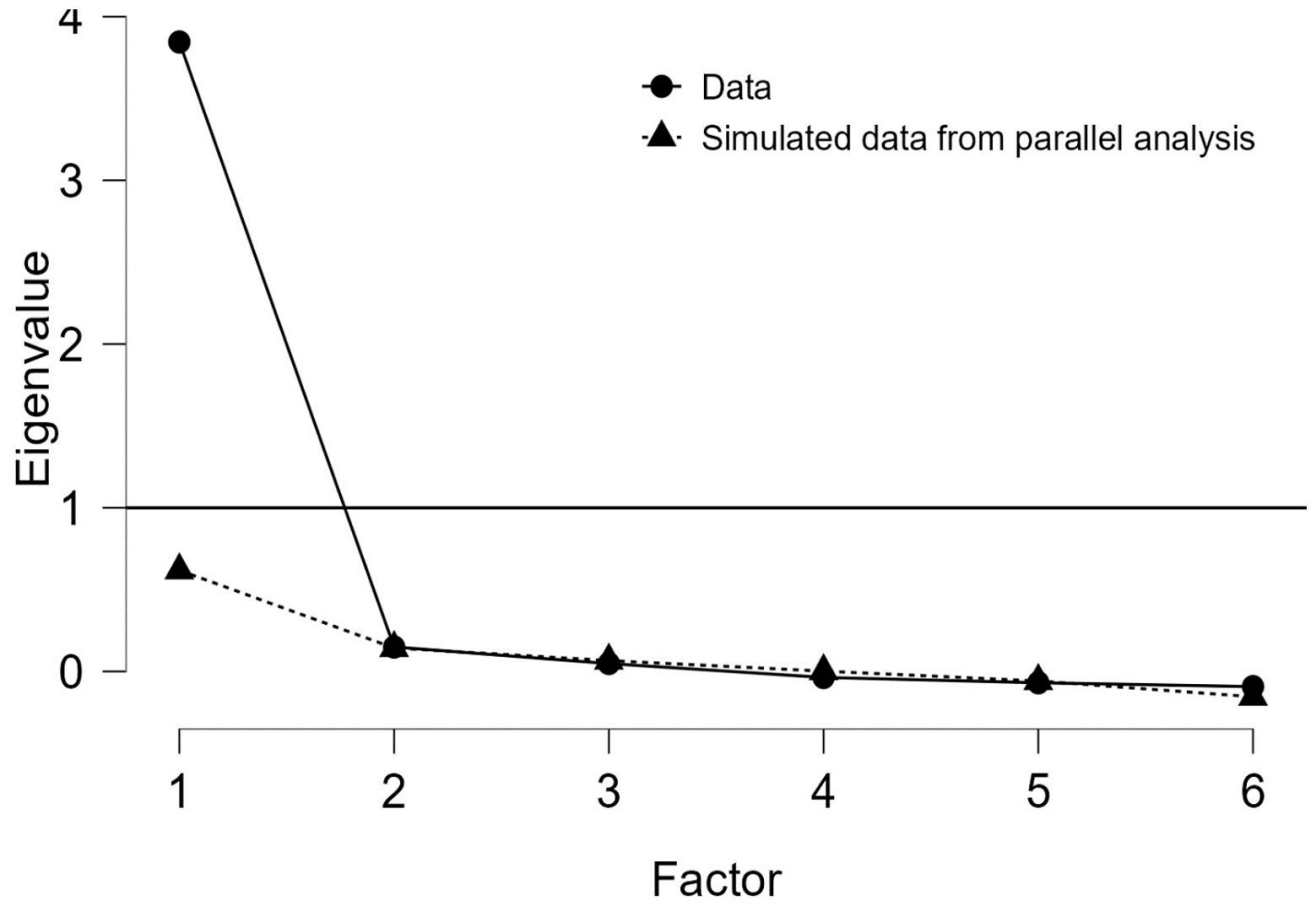
Scree plot and parallel analysis of observed and simulated eigenvalues.

Exploratory factor analysis was performed on the first sample (*n* = 220). In line with the original study (McKenna-Plumley et al., 2024), Parallel Analysis (PA) was conducted using principal axis factoring with oblimin rotation, as it is theoretically assumed that all factors of existential loneliness are correlated with each other. The results of the Kaiser-Meyer-Olkin measure (KMO = 0.9) and the significant Bartlett’s test of sphericity (χ^2^(15) = 847.39, *p* < 0.001) indicate the adequacy of the sample correlation matrix for statistical analysis. The PA results according to the Horn criterion suggested the presence of one factor, with an eigenvalue of 3.85, explaining 64.1% of the variance. The other factors had eigenvalues less than 0.54. All item loadings on this factor were greater than 0.72 (0.72–0.87), so all items were retained, indicating the unidimensionality of the Arabic version of BSEL. Figure 1 visually illustrates the scree plot from the parallel analysis.

### 3.3 Results of the confirmatory factor analysis

**Figure 2:**
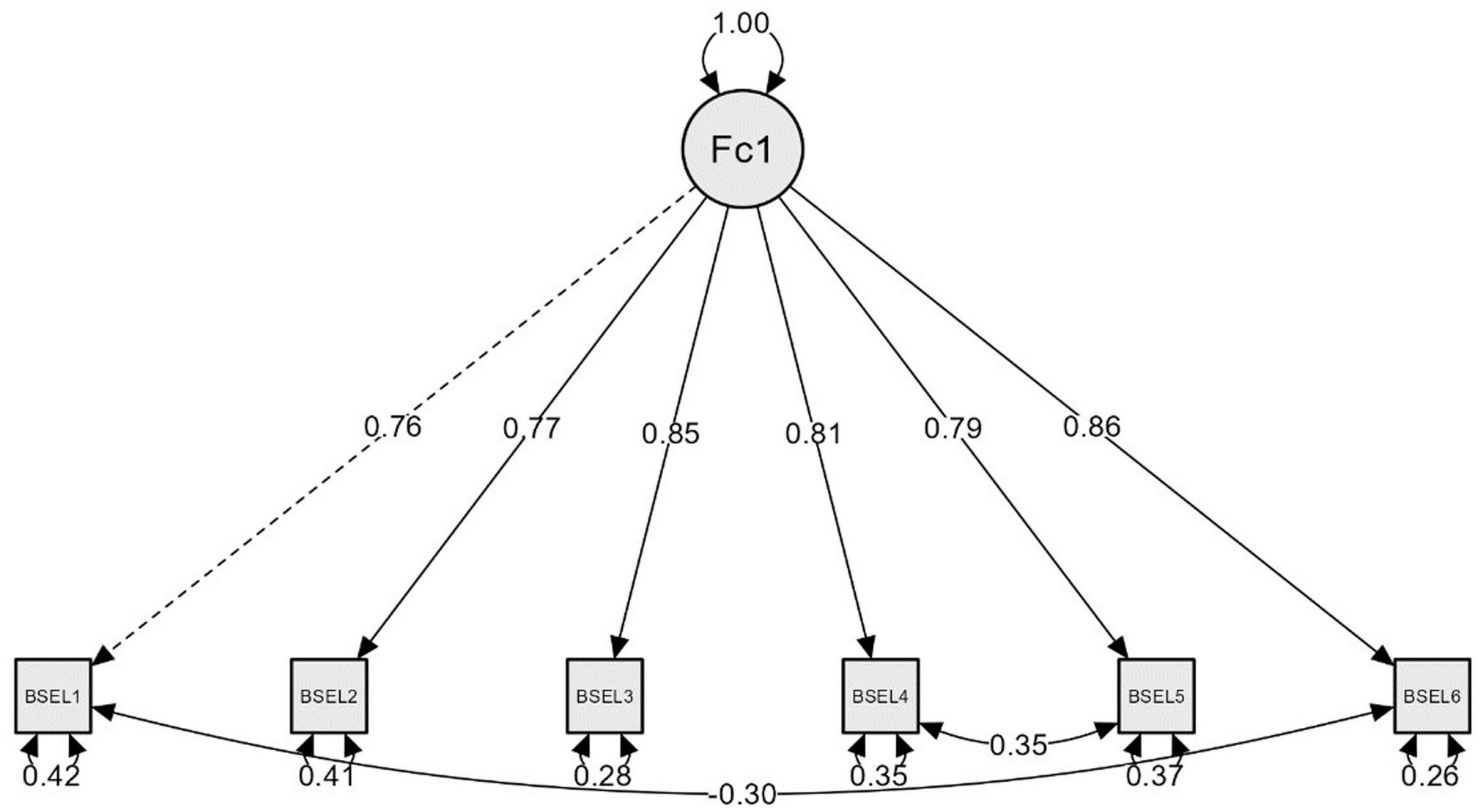
Results of confirmatory factor analysis for the Arabic version of BSEL.

Confirmatory factor analysis was performed to assess the unidimensional structure derived from the exploratory component analysis on the second sample (*N* = 200). The KMO value was 0.913, and Bartlett’s test of sphericity confirmed the data’s adequacy for factor analysis (χ^2^(15) = 807.1, *p* < 0.001). The initial analysis revealed weaknesses in the model fit indices, X^2^(9) = 35.54; X^2^/df = 3.95, *p* < 0.001; CFI = 0.97; TLI = 0.95; SRMR = 0.031; RMSEA = 0.12 (90% CI, 0.081–0.12), as these indices failed to fulfill all necessary criteria. Examination of the model modification indices suggested that correlating the covariance errors of item pairs (4, 5) and subsequently (1, 6) would enhance the model fit indices, due to the semantic similarity between the inability to communicate with others (item 4) and being separated from everyone (item 5). There exists a semantic equivalence between the terms “separation” (item 1) and “loneliness” (item 6). This approach provided a noticeable enhancement in the model indicators: X^2^(7) = 12.05, X^2^/df = 1.72, CFI = 0.99, TLI = 0.99, SRMR = 0.019, and RMSEA = 0.06 (90% CI, 0.0–0.12). All factor loadings exceeded 0.76. Table 2 presents the factor loading values (ß), means, and standard deviations for the Arabic version of the BSEL scale items. Figure 2 graphically represents the results of confirmatory factor analysis for the Arabic Version of BSEL.

### 3.4 Measurement invariance

An MG-FCA analysis was performed on the entire study sample (*n* = 420) to assess measurement invariance across gender and age. Age was categorically divided into two groups: 18–35 years for young adults and 36–82 years for older adults, based on the characteristics of the age distribution in the present sample (mean age). A series of MG-FCA were subsequently conducted to confirm the invariance across the four levels: structural, metric, scalar, and residual. The configural model showed an adequate fit with the study data regarding gender. Imposing additional constraints on the factor loadings to assess metric invariance did not result in a significant change in model fit, indicating the achievement of metric invariance. Correspondingly, scalar invariance was established, as changes in fit indices remained within permissible limits. Moreover, residual invariance was established with minor changes in model fit, indicating equal residual errors and exhibiting full measurement invariance across genders. Regarding measurement invariance across age groups, the configural invariance showed acceptable fit, while metric and scalar invariance were established, as the changes in fit indices were minor within the established criteria. However, the residual invariance model showed a notable decline in model fit, suggesting insufficient support for residual invariance, which was deemed adequate. Table 3 details these results.

**Table 3:** Measurement invariance of the Arabic version of BSEL across gender and age groups.

Model	Fit statistics					CFI	ΔCFI	RMSEA	ΔRMSEA	SRMR	ΔSRMR
	χ^2^	df	Δχ^2^	Δdf	Χ^2^_/_df						
Gender											
Configural	36.997	14			2.64	0.986		0.088		0.025	
Metric	42.927	19	5.93	5	2.259	0.986	0.000	0.077	−0.011	0.045	0.020
Scalar	51.881	24	8.954	5	2.16	0.983	−0.003	0.074	−0.003	0.044	−0.001
Strict	67.921	32	16.04	8	2.121	0.979	−0.004	0.073	−0.001	0.041	−0.003
Age											
Configural	24.676	14			1.74	0.994		0.060		0.020	
Metric	30.810	19	6.134	5	1.622	0.993	−0.0001	0.054	−0.006	0.042	0.022
Scalar	35.710	24	5.278	5	1.284	0.993	0.000	0.048	−0.006	0.039	−0.003
Strict	110.912	32	75.202	8	3.466	0.954	−0.039	0.108	0.065	0.053	0.014

df, degrees of freedom; CFI, comparative fit index; RMSEA, root mean square error of approximation; SRMR, standardized root mean square residual.

### 3.5 Reliability

Reliability of the Arabic A-BSEL version was examined to assess the internal consistency of its items, using Cronbach’s *α* and McDonald’s *ω*. The whole study sample has a reliability coefficient of ω = 0.92 and α = 0.92. In the first sub-sample (ω = 0.91, α = 0.91). For the second sub-ample (ω = 0.92, α = 0.92). These values indicate that the scale has a high degree of internal consistency and reliability. The scale’s reliability was also assessed using the test–retest method over a three-week interval, yielding a value of *r* = 0.76. This result indicates that the scale has a sufficient level of score stability over time.

### 3.6 Convergent and divergent validity

Convergent and divergent validity of the Arabic version of the A-BSEL scale were evaluated by assessing correlations (Pearson correlation) between the scale scores and other related psychological variables (existential isolation, depression, and happiness) in the first sub-sample (*N* = 220). The A-BSEL scores exhibited a strong positive correlation with existential isolation (*r* = 0.63, *p* < 0.01) and depressive symptoms (*r* = 0.62, *p* < 0.01), thereby indicating convergent validity and suggesting conceptual convergence between the constructs; however, it did not meet the criteria for conceptual redundancy as outlined by Cohen (1988). The scale showed a negative correlation with happiness (*r* = −0.45, *p* < 0.01), thereby supporting its discriminant validity by differentiating it from the expressions of positive emotional functions. Table 4 shows these results. The discriminant validity of the BSEL scale was also examined using the HTMT ratio indicator between the latent dimensions of all constructs (BSEL, existential isolation, happiness, and depression). The results indicated that the HTMT values ranged between 0.362 and 0.684, all of which were below the specified threshold of 0.85, indicating good discrimination between the latent constructs (Henseler et al., 2014; Cheung et al., 2023). The correlations between BSEL, on one hand, and existential isolation, happiness, and depression, on the other hand, ranged between 0.532 and 0.684, and the HTMT value between BSEL and depression remained below the proposed threshold (0.85).

**Table 4:** Correlation matrix between the BSEL existential loneliness scale, related measures, and age.

Measures	1	2	3	4	5
Brief Existential loneliness scale (BSEL)	-				
Existential isolation scale (EIS)	0.63^**^	-			
Depression Anxiety Stress Scale (DASS-21)	0.62^**^	0.41^**^	-		
The Arabic scale of Happiness (ASH)	−0.45^**^	−0.35 ^**^	−0.32^**^	-	
Age	−0.11^*^	−0.04	−0.11	−0.06	-

^**^p > 0.01; ^*^p < 0.05.

### 3.7 Differences in existential loneliness means on the A-BSEL scale across the gender and age groups

A t-test for independent samples was used to examine differences in existential loneliness between males and females based on the total score in the whole sample. The results showed a statistically significant difference between the two groups (d = 0.26, *p* < 0.001, t (418) = −2.56). The effect size estimated using Cohen’s d suggests that the difference is small in practical terms. In terms of age, there were no significant differences between the study’s age groups (18–35 and 36–80 years), t (418) = 1.39, (*p* = 0.16). These results are recorded in Table 5.

**Table 5:** t-retest result of comparison BSEL scores by gender and age groups.

variable	N	M	SD	t	*p*	Cohen’s d
Gender						
male	149	10.66	4.83	−2.56	0.011	0.26
female	271	11.89	4.63			
Age group*						
Group 1	232	11.57	4.89	0.538	0.591	0.053
Group 2	188	11.32	4.54			

*Group 1 = age: 18– ≤ 35 years, Group 2 ≥ 36 year. M, mean; SD, standard deviation.

## 4 Discussion

This study has introduced a new scale for existential loneliness for the first time in the Arabic existential psychological literature, which researchers can use for cross-cultural investigations and surveys. We aimed to develop a scale that effectively assesses this dimension of loneliness, which has seen heightened interest globally over the last two decades in both qualitative and quantitative research. Our goal was to translate the Brief Existential Loneliness Scale (BSEL) and assess its psychometric properties and factor structure within an Eastern culture that differs greatly from Western culture. It is a collectivist culture where religiosity profoundly affects human existence, in contrast to the secular, individualistic culture that prevails in the Western societies where the original scale was developed. Religion may play a complex role in shaping the experience of existential loneliness; on one hand, it contributes to enhancing the sense of meaning and purpose in life, which aligns with the existential perspective that the availability of meaning alleviates feelings of emptiness and loneliness (Frankl, 1984). It may provide effective strategies for coping with stress, enhancing social support and belonging, serving as a source of psychological adaptation, thereby contributing to the reduction of existential loneliness (Pargament, 1997). On the other hand, religiosity based on guilt or religious conflict may contribute to increased psychological distress and deepen negative existential experiences (Pargament et al., 2000). Accordingly, we can initially conclude that the impact of religiosity on existential unity in Arab societies does not appear to be linear, but rather is determined by the nature of religiosity and its integration with the individual’s psychological and social structure within the cultural context. We selected this scale because of its empirical validation and robust theoretical framework. The most challenging and precise step of the current study was the adaptation to Arabic, considering the previously indicated cultural differences. Therefore, the effort to convey existential psychological meanings in the simplest way to capture this phenomenon was an enormous challenge. We were seeking to follow optimal guidelines in cross-cultural scale adaptation, avoiding literal translation of the items. We verified that the content of the items aligned with the respondents’ current values and views to prevent non-responsiveness. The final translated version of BSEL was appropriate for all native Arabic speakers, regardless of their location.

The rationale for employing exploratory factor analysis in the present study was consistent with established best practices for adapting psychological measures to new settings. Furthermore, it was essential to verify that the disparities in cultural frameworks between Western and Eastern cultures are not affecting the concept of existential loneliness as a universal construct, as defined in existential literature (e.g., Chung et al., 2020). This is what the EFA results clarified about the unidimensionality of the BSEL scale, as in the original study (McKenna-Plumley et al., 2024). It indicates a strong similarity in the concept of existential loneliness between the present study population and the original study, since one factor explained about 65% of the variance in EL, comparable to 71% in the original BSEL. The CFA results validated the exploratory study’s finding that the Arabic translated version of A-BSEL is unidimensional.

It is worth noting that there is a difference in the modification indices between the two versions of the BSEL. Correlating the residual errors of two pairs of items (4, 5) and (1, 6) that are similar either in words or implicit meaning sufficiently enhances the model’s fit with the current data. Conversely, correlating the residual errors of a single pair of items (2, 5) in the original scale enhances the fit of the one-factor model. The reason may lie in an individual’s comprehension and interpretation of items, influenced by linguistic characteristics and cultural factors. For example, item 4, “suffering from a feeling of inability to fully communicate with others,” corresponds to “the painful feeling of separation from everyone” (item 5), meaning that the inability to communicate with others (item 4) is similar to and/or leads to separation from everyone (item 5). Likewise, in the pair of items (1, 6), the term “separation” (item 1) is equivalent to “loneliness” (item 6). In general, this result indicates that the construction of existential loneliness as measured by A-BSEL is similar to what the original BSEL measures. All these items convey a similar implicit negativity, as described by McKenna-Plumley et al. (2024). Allowing the addition of a covariance between the measurement errors of these items improved the final model’s fit to the data. The initial model did not meet all criteria for goodness of fit, especially the RMSEA value. In structural equation modeling, the literature supports correlating measurement errors in this case. There is clear theoretical justification because of the items’ similar linguistic formulation, shared context, and method effects. These changes reflect specific characteristics of the elements within the cultural and linguistic context of the Arabic scale, and were not made arbitrarily. Regarding reliability, in addition to the high internal consistency indicators, the current study is distinguished by an assessment of the scale’s score stability (*r* = 0.76), which suggests the stability of A-BSEL results over time. These findings extend previous research by providing the first evidence of the scale’s temporal stability using a test–retest method across a three-week interval between the first and second administrations. The strong positive correlations between A-BSEL scores and existential isolation (*r* = 0.63) suggest a strong theoretical correlation between EL and EI, although it does not extend to conceptual overlap. It implies a conceptual distinction from existential isolation, although they have a shared existential core. This finding is consistent with McKenna-Plumley et al. (2024). It is also relatively consistent with Pinel et al. (2017), who found a significant correlation between EL and EI, but with a lower value (*r* = 0.28). The results also revealed that A-BSEL scores were positively correlated with depression (*r* = 0.62), indicating a shared variance between EL and depressive symptoms. This aligns with the findings of McKenna-Plumley et al. (2023) that existential loneliness is not a transient condition but rather is involved in the psychological mechanisms associated with depression, which is also consistent with previous studies that indicated existential loneliness as predicting depressive symptoms (Gökdemir-Bulut and Bozo, 2018; Mayers et al., 2002). However, it also suggests that the BSEL can capture existential loneliness as a distinct construct. These results indicate the convergent validity of the A-BSEL construct. In contrast, the significant negative correlation of A-BSEL with happiness indicates the scale’s ability to distinguish existential loneliness as a negative trait from happiness as a positive trait, thereby validating the scale’s discriminant validity. In general, these associations suggest that existential loneliness is a fundamental psychological variable in understanding distress (Mayers et al., 2002; Pinel et al., 2017; Helm et al., 2020). The HTMT analysis revealed that existential loneliness, as measured by BSEL, is empirically distinct from depressive symptoms and is not simply a repetition of them, but rather a conceptually separable dimension that cannot be reduced to general psychological distress (Henseler et al., 2014). The significant negative correlation between existential loneliness and age has revealed an inverse relationship that indicates younger adults experiencing higher levels of existential loneliness compared to older adults. This finding is consistent with the findings reported by McKenna-Plumley et al. (2024). Therefore, attention should be directed to this age group, given the consequences of existential loneliness, as it is related to depression (Helm et al., 2020) and has implications as a predictor of psychological disorders (Álvarez et al., 2023; Fu et al., 2023). We emphasize that these findings are preliminary, given the weak correlation coefficient obtained in this study and the absence of research so far on these two variables. Furthermore, this finding appears contradictory to other qualitative research that has shown the elderly as being more prone to existential loneliness as they age and near the end of their lives (e.g., Edberg et al., 2023; McKenna-Plumley et al., 2023). Although age is connected with existential loneliness and is regarded a moderating element, it is not the only determining factor. According to relevant literature, age and loneliness follow a U-shaped curve, with loneliness increasing in early youth and old age and decreasing in middle age (De Witte et al., 2025; Graham et al., 2024). Furthermore, the characteristics of existential loneliness fluctuate between age groups. In youth and early adulthood, problems of identity and feelings of liminality emerge (Garnow et al., 2022), but in old age, aspects of role loss, disease, and confronting mortality become more important (Olofsson et al., 2021). Regarding the impact of individual versus collectivist cultures on loneliness, psychological literature shows varied results. Some studies suggest that average feelings of loneliness are higher in individualistic cultures and lower in collectivist ones (Barreto et al., 2021). In contrast, other studies find higher loneliness levels in more collectivist societies (e.g., Luhmann et al., 2022). This phenomenon is called the cultural paradox of loneliness: in individualistic cultures, freedom and isolation increase loneliness risk, whereas, in collectivist cultures, strict norms and high expectations raise loneliness risk by heightening the sense of failure or non-belonging (Rokach, 2018). It is also notable that people in collectivist cultures perceive higher societal stigma around loneliness because social integration failure is seen as a more significant personal flaw or deviation from group norms than in individualistic societies (Barreto et al., 2022; Luhmann et al., 2022). Thus, using a scale like BSEL in a collectivist society, such as Arab communities, may lead respondents to interpret items as threats to group status or as evidence of non-belonging, even in social environments. Therefore, researchers must consider differences in stigma and their impact on scale accuracy when applying measures developed in individualistic contexts to collectivist ones. This work is the first study that assessed the measurement invariance of the BSEL. Strict measurement invariance by gender was attained, indicating that factor loadings, item intercepts, and residual covariance on the Arabic version of the BSEL are equal for both men and women. Consequently, the observable and latent differences between the two groups could be interpreted as genuine differences rather than just measurement errors. This finding aligns with other studies on comparable existential measures, such as the Existential Loneliness Scale (Hadeei, 2023), which revealed no differences in performance (DIF) based on gender. Measurement invariance across age groups has been established at the level of scalar invariance, which is sufficient for dependable group comparisons (Putnick and Bornstein, 2016). No prior research has investigated measurement invariance across age groups for existential scales; thus, these findings are deemed preliminary. When comparing the mean scores on the total BSEL score between the two age groups, the 18–35 age group had higher mean scores than the group over 35, without significant differences. This calls for attention and adequate care for this group, and this result also aligns with what was revealed by McKenna-Plumley et al.’s (2024) study. Clinically, achieving measurement invariance at the scalar level allows comparison of mean levels of existential loneliness between age groups with reasonable confidence. This also supports using the scale to monitor differences between adults and older adults (Panayiotou et al., 2021; Xiao and Du, 2023). The scale can be used in screening to identify individuals who need a more in-depth assessment or intervention for distress related to loneliness across age groups (Senese et al., 2021). Generally, achieving scalar invariance is sufficient for routine clinical and research use (Alsubheen et al., 2021). Exercise caution when making precise interpretations of very small differences between age groups, since a lack of equal residual errors in the absence of strict invariance can add a bit of repetition to the observed scores (Manera et al., 2021). Regarding gender differences, the t-test revealed a statistically significant difference between the scores of males and females regarding existential loneliness, with a small effect size in favor of female. The interpretation of these differences is based on a robust methodology in light of the full measurement invariance results as established by MG-CFA, which makes the comparison of means by gender statistically valid. The observed differences in BSEL scores reflect real differences, not just resulting from item interpretation variations due to gender or measurement bias. Accordingly, we can treat these differences as valid, noting however that the small effect size may reduce their practical significance, which calls for caution when generalizing the results. The Arabic version of the BSEL scale provides an important basis for identifying groups most susceptible to psychological distress and enhances early preventive efforts (Van Orden et al., 2010). The literature on loneliness indicates that integrating a measure of existential loneliness into clinical assessment helps uncover the deeper dimensions of psychological distress associated with self-isolation and loss of meaning, which traditional measures of loneliness may not adequately capture (Pinel et al., 2017). At the therapeutic level, diagnosing existential loneliness contributes to guiding and improving meaning-based interventions within the framework of existential therapies, which are noted for their effectiveness in reducing feelings of loneliness and enhancing psychological well-being (Wong, 2010; Vos et al., 2015). Therefore, offering a practical and concise tool for measuring existential loneliness represents an added value in integrated clinical practice.

### 4.1 Limitations and future studies

Despite the strengths of this study’s results, being the first adaptation of an existential loneliness tool for native Arabic speakers, it is not without some limitations related to the relatively small sample size and the method of data collection via the internet, which may introduce bias toward participants with higher digital literacy. Additionally, it does not represent of other ethnicities such as Circassians and Chechens or other non-Muslim religious sects within Jordanian society. Furthermore, the study’s reliance on self-report measures may expose the results to social desirability bias or response biases. Moreover, the sampling method using a snowball sample may limit the sample’s representation and reduce the ability to generalize the results to a broader population. Therefore, caution should be exercised when generalizing the results beyond the study sample. The sample also did not include frail elderly individuals or people known for the recurrent deterioration of their mental health compared to ordinary individuals as a criterion for the scale’s discriminative validity. We encourage comprehensive research involving older adults in residential care facilities, patients with severe illnesses such as cancer or those undergoing dialysis, and teenagers, particularly during the transition from adolescence to adulthood. Future studies should use randomized designs and diverse data sources to verify the validity of the results and enhance their generalizability.

## 5 Conclusion

The Arabic-adapted version of the Brief Existential Loneliness Scale is an easy-to-apply, brief instrument with favorable psychometric properties. The construction and adaptation were based on a robust standard, starting with the translation of the scale into Arabic and encompassing the associated steps and stages, leading to exploratory and confirmatory experiments that demonstrated a unidimensional structure aligned with the original development, grounded in a strong theoretical framework. Furthermore, the scale has adequate construct validity evidence, indicating its potential for use in scientific research to survey the phenomenon of existential loneliness or as a complement in screening alongside general loneliness scales for professionals in the field of cognitive-behavioral therapy.

## Data Availability

The datasets presented in this study can be found in online repositories. The names of the repository/repositories and accession number(s) can be found at: https://doi.org/10.17605/OSF.IO/GYA2H.
